# Abnormal Dosage Compensation of Reporter Genes Driven by the Drosophila Glass Multiple Reporter (GMR) Enhancer-Promoter

**DOI:** 10.1371/journal.pone.0020455

**Published:** 2011-05-31

**Authors:** Corey Laverty, Fang Li, Esther J. Belikoff, Maxwell J. Scott

**Affiliations:** Institute of Molecular BioSciences, Massey University, Palmerston North, New Zealand; University of Texas MD Anderson Cancer Center, United States of America

## Abstract

In *Drosophila melanogaster* the male specific lethal (MSL) complex is required for upregulation of expression of most X-linked genes in males, thereby achieving X chromosome dosage compensation. The MSL complex is highly enriched across most active X-linked genes with a bias towards the 3′ end. Previous studies have shown that gene transcription facilitates MSL complex binding but the type of promoter did not appear to be important. We have made the surprising observation that genes driven by the glass multiple reporter (GMR) enhancer-promoter are not dosage compensated at X-linked sites. The GMR promoter is active in all cells in, and posterior to, the morphogenetic furrow of the developing eye disc. Using phiC31 integrase-mediated targeted integration, we measured expression of *lacZ* reporter genes driven by either the GMR or *armadillo* (*arm*) promoters at each of three X-linked sites. At all sites, the *arm-lacZ* reporter gene was dosage compensated but *GMR-lacZ* was not. We have investigated why GMR-driven genes are not dosage compensated. Earlier or constitutive expression of GMR-lacZ did not affect the level of compensation. Neither did proximity to a strong MSL binding site. However, replacement of the *hsp70* minimal promoter with a minimal promoter from the X-linked *6-Phosphogluconate dehydrogenase* gene did restore partial dosage compensation. Similarly, insertion of binding sites for the GAGA and DREF factors upstream of the GMR promoter led to significantly higher *lacZ* expression in males than females. GAGA and DREF have been implicated to play a role in dosage compensation. We conclude that the gene promoter can affect MSL complex-mediated upregulation and dosage compensation. Further, it appears that the nature of the basal promoter and the presence of binding sites for specific factors influence the ability of a gene promoter to respond to the MSL complex.

## Introduction

The *Drosophila* eye is well suited to genetic investigations, as it is a dispensable tissue with mutant phenotypes that are relatively easy to identify [1]. Glass is a Zinc-finger transcription factor involved in photoreceptor development, present in the eyes, ocelli, small areas of the brain, and the embryonic Bolwig organ [2], [3]. The glass multiple reporter (GMR) is a pentamer of a 27 bp Glass response element from the *Rh1* (*ninaE*) promoter, upstream of a minimal promoter from the *hsp70* gene [4]. The GMR enhancer-promoter drives expression in all cells in, and posterior to, the morphogenetic furrow in the developing eye imaginal disc [5], [6]. Expression of the pro-apoptotic *hid* gene from GMR kills eye cells proportional to the *GMR-hid* expression level; a smaller eye with higher levels [7]. This reporter system facilitated a mutational screen for apoptosis-related factors, by identifying modifiers of *GMR-hid*-derived eye size [8]. We sought to use the variable eye size of *GMR-hid* to screen for modifiers of *Drosophila* dosage compensation, but observed that *GMR-hid* did not report on dosage compensation. We have thus investigated why *GMR-hid* failed to respond, and report that the minimal *hsp70* promoter appears refractory to the compensation machinery.

*Drosophila* males (XY) lack a dose of X-linked genes in comparison to (XX) females, but compensate for this by doubling transcription from the single X chromosome [9], [10], [11]. Dosage compensation requires the action of the RNA-containing Male-Specific Lethal (MSL) complex, which cannot form in females as translation of *msl2* RNA is repressed by the female-specific SXL protein [12]. The MSL complex specifically binds to many sites on the male X chromosome and modifies the chromatin, most notably by acetylating histone H4 at lysine16 (H4K16) [13]. The enrichment for H4K16ac across active genes could lead to a less compact chromatin structure as incorporation of H4K16ac into nucleosomal arrays abolished a salt-dependent compaction into 30 nm-like fibres in an *in vitro* assay [14]. However, it has been reported that H4K16ac is more strongly associated with DNA replication timing than transcription in *Drosophila* cells [15]. Thus the exact mechanism of transcription enhancement by the MSL complex is unknown. It has been suggested that the MSL complex may enhance transcription elongation as the MSL complex is enriched on active genes with a bias towards the 3′ end [16], [17], [18], [19]. Indeed, using a global run-on sequencing (GRO-seq) approach, Kuroda and colleagues concluded that the MSL complex enhances transcription by facilitating the progression of RNA polymerase II along X-linked gene [20].

The dosage compensation machinery can also affect genes that are not endogenous to the X chromosome. Translocations of genomic fragments between the X chromosome and autosomes exhibit “spreading” of the MSL complex from the X-linked sequences into the adjacent autosomal regions [21]. This ability of the MSL complex to bind autosomal genes is likely the reason why autosomal genes translocated to the X chromosome usually gain increased transcription in males [22], [23], [24]. Short sequences with strong affinity for MSL complex can recruit the complex to autosomes, and increase the transcription of neighbouring genes [25], [26], [27], [28]. These MSL-binding High Affinity Sites (HAS) are thought to serve as primary attractors of the MSL complex to the X chromosome, from which it can then “spread” to affect the transcription of nearby active genes [29]. Indeed, chromosome-wide studies of MSL binding find that most MSL-bound genes are active [17]. Thus, we expected the (active) *GMR-hid* to respond to dosage compensation, but found that it was unable to fully do so. We argue that an active gene is not sufficient for recognition by the dosage compensation machinery, even if near a HAS, and specifically that the *hsp70* minimal promoter appears to hinder dosage compensation.

We also report two further observations of oddities in expression from GMR that will be of interest to those working with this enhancer-promoter. Activity of beta-galactosidase produced by a *GMR-lacZ* transgene was not linear with respect to gene dose, at least in the environment of the female X chromosome. The activity in females with two copies of the transgene was more than twice the activity in single-copy females, suggesting some synergy in homozygosity. Further, we detected some repression of expression from GMR in the larval imaginal tissue, consistent with that first reported for an earlier Glass-based eye reporter that contained a longer glass response element [30]. Both these observations have implications for dosage compensation, but in general they also call for careful use of the GMR enhancer-promoter.

## Results

### *GMR-hid* is a poor reporter of dosage compensation

Our understanding of the mechanism of X chromosome dosage compensation may need to better appreciate the balance between the MSL complex and the wider network of chromatin regulators. Identification of all factors that play a role in dosage compensation is not trivial. The five MSL proteins (MSL1, MSL2, MSL3, MLE, and MOF) were identified based on mutant phenotypes of male-specific lethality [31], [32]. Loss of function mutations in genes for more generally acting epigenetic modifiers would be expected to be homozygous lethal for both sexes. However, some factors have been implicated to have a role in dosage compensation based on hypomorphic phenotypes of reduced male viability or altered male X chromosome morphology [10]. Such factors include the DNA supercoiling factor [33], JIL-1 histone kinase [34] and the heterochromatin-associated protein SU(VAR)3–7 [35]. The initial aim of this study was to search for novel dosage compensation factors, not by screening for male-lethality, but by using the reduced eye size of *GMR-hid* to screen for mutations that affected the end product of dosage compensation: the equalization of phenotype.

**Figure 1:**
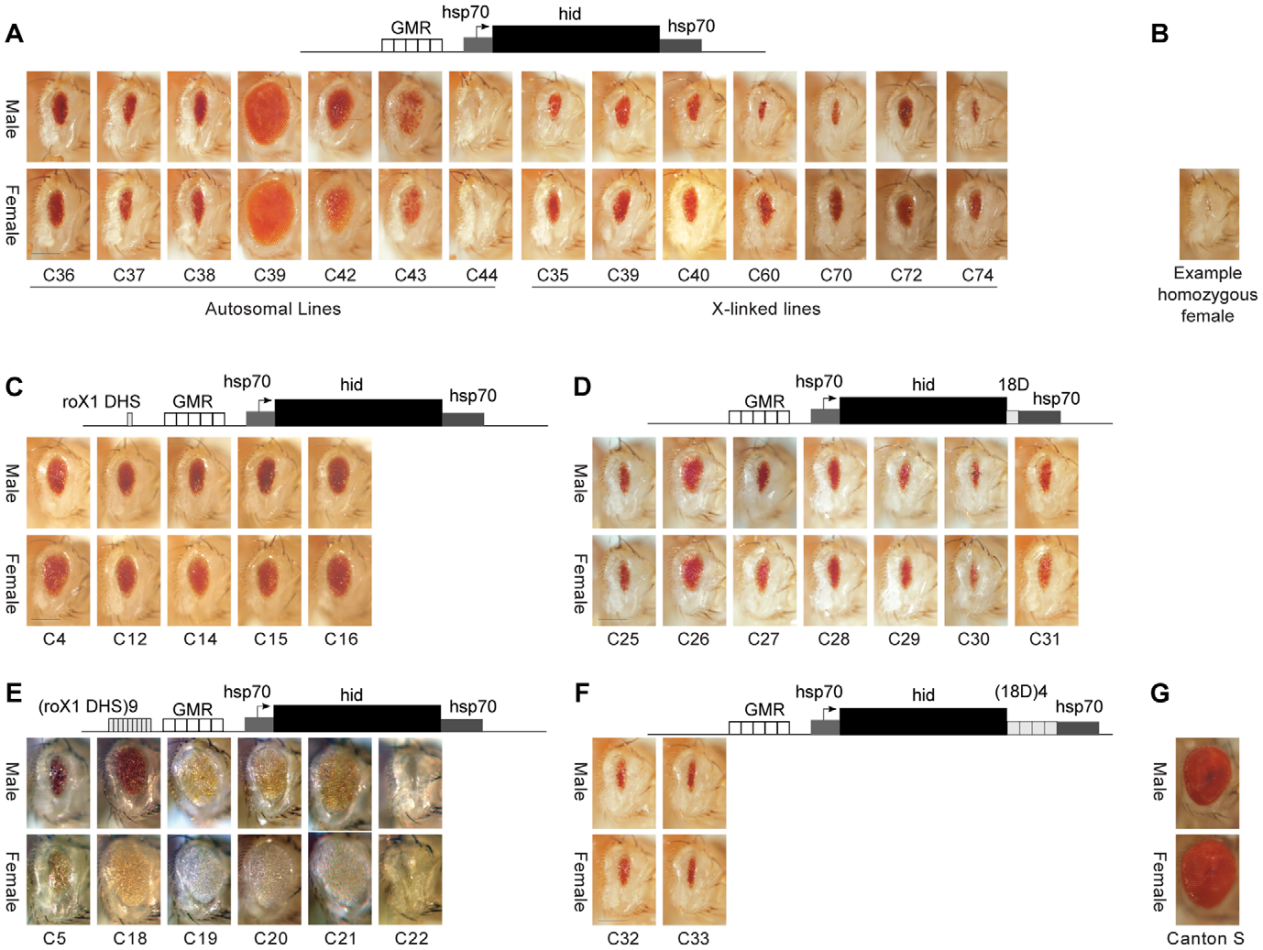
*GMR-hid* constructs were expressed similarly in both sexes. A, C–F) *Drosophila* eyes from transgenic lines (e.g. C32) carrying one copy of the indicated constructs. A) Lines C70, C72 and C74 are phi-C31 mediated insertions at the attP sites 2A, 6E and 20C respectively. B) An example eye from a female homozygous for *GMR-hid* construct. G) Eye from wild-type (Canton S). In all panels right eyes are shown, dorsal up, anterior right. Bar  = 0.2 mm.

We followed two strategies to adapt the *GMR-hid* system to report on *Drosophila* dosage compensation, with the aim of then conducting a mutagenesis screen for modifiers of dosage compensation. To obtain a sex-specific difference in eye size, we created *P*-element transgenic lines carrying *GMR-hid*, and screened for those genetically linked to the X chromosome (Figure 1A). Transgenic insertions on the X chromosome usually acquire a degree of compensation [22], [23], [24], meaning that males with one transgene copy express more than single-copy females, and perhaps as much as two-copy females. In all cases, flies homozygous for the *GMR-hid* transgene had ablated eyes (Figure 1A **)** representing high levels of transgene expression, but little information as to what level of expression. All lines homozygous for any *GMR-hid* transgene were weak, presumably due to leaky expression of *hid* from the basal *hsp70* TATA, or expression in other neuronal tissues. Flies heterozygous for all autosomal *GMR-hid* insertions had similar eye sizes in each sex (Figure 1B). Surprisingly, lines with X-linked insertions of *GMR-hid* also showed very little difference in eye size between the sexes. In most cases, (hemizygous) male eyes were only slightly smaller than heterozygous female eyes; both sexes having a small range of sizes that just overlapped. The line with greatest difference and least overlap between the sexes (line C60) was selected for further analysis.

To validate the usefulness of the line in a mutagenesis screen, we crossed line C60 to flies deficient in *msl1*, *msl2*, and *mle*. Flies heterozygous for both *GMR-hid* and each *msl* deficiency (*GMR-hid/+; msl1 msl2 mle/+*) had similar sized eyes to those without the *msl* deficiency (Figure S1), indicating an inability to report on mutations in known MSL components. As an alternative genetic test to determine if *GMR-hid* could respond to the MSL complex, we crossed line C60 to a line that constitutively expresses MSL2. Females with *hsp83-msl2* can assemble MSL complex, erroneously up-regulate X-linked gene expression, and thus have poor viability [36]. However, *GMR-hid/+; hsp83-msl2/+* females were identical to females that lacked the *hsp83-msl2* transgene (Figure S1), indicating that the sex-specific eye size may not have reflected action by the MSL complex. The strategy seemed unsuitable for the proposed mutagenic screen.

In a complementary approach, we sought to directly attract MSL complex to autosomal insertions of *GMR-hid* by coupling the transgene to the strongest known MSL binding sites. The MSL complex binds with high affinity to a DNase I hyper-sensitive site (DHS) in the X-linked *roX1* gene [26]. The long *roX1* noncoding RNA is a component of the MSL complex. *roX1* expression is tightly regulated through a combination of constitutive repression and male-specific hyper-activation by the MSL complex that antagonises the repression [37]. Ectopic autosomal insertions of the *roX1* DHS attract significant amounts of MSL complex to the insertion site, clearly visible by immuno-fluorescent labelling of polytene chromosomes, and a nine-copy multimer of the DHS is particularly effective [26]. The *roX1* DHS is also capable of mediating increased activity of a coupled *lacZ* reporter gene at autosomal insertions [25].

We created *roX1 DHS-GMR-hid*, and *(roX1 DHS)9-GMR-hid*, and observed the eye phenotype in flies carrying autosomal insertions of each (Figure 1C,E). Lines carrying single insertions of *roX1-DHS-GMR-hid* had eyes of equal size in each sex. Those with the nine-copy multimer of the binding site displayed a range of eye sizes, mostly larger than for *roX1 DHS-GMR-hid*, with male eyes in most cases slightly smaller than female eyes. Thus, eye size did not respond well to any MSL complex recruited by the *roX1 DHS*.

The MSL complex binds the X chromosome most strongly at hundreds of MSL Recognition Elements (MREs), GA-rich motifs that are found predominantly in intergenic or intronic sequences [38], [39]. The MRE at cytological position 18D11 has strong affinity for MSL complex, and can attract the complex to an ectopic autosomal insertion site, especially when multimerized [40]. We placed both the MRE from 18D11 and the tetramer of the site in the 3′ UTR of *GMR-hid*, to mimic the normally high concentrations of MSLs towards the 3′ end of a transcript. However, flies transgenic for either construct still displayed eyes of similar size in males and females (Figure 1 D, F). It appeared that directly attracting MSL complex to *GMR-hid* did not improve the ability of the reporter to respond to dosage compensation.

The failure of *GMR-hid* to report might have been due to an inability to report two-fold transcriptional changes as an altered eye size. However, we observed a wide range of possible eye sizes (that likely reflected position effects on transgene expression), and the system has displayed great sensitivity previously [8]. Further, the ablated eye phenotype of two copy females showed that the system was dose-responsive. Instead, we theorized that *GMR-hid* was not amenable to dosage compensation. An alternative, although unlikely explanation, was the MSL complex was not recruited to the transgenes containing a *roX* or 18D11 high affinity site. Yet, in *(roX1 DHS)9-GMR-hid* lines (Figure 1 E) female eye color was generally lighter than males, and in some cases indistinguishable from white. Additionally, there was variegation in pigmentation in some lines (e.g. C20, C21). This is consistent with general repression of the mini-*white* transformation marker gene, responsible for the red pigment, with male-specific relief by the MSL complex, as previously observed at some autosomal insertions of mini-*white* when coupled to a *roX1* transgene [41]. This suggests the MSL complex was recruited to the multimer of the *roX1* DHS, sufficient to relieve repression of mini-*white*, but with little effect on *GMR-hid* expression.

### The promoter can affect compensation

**Figure 2:**
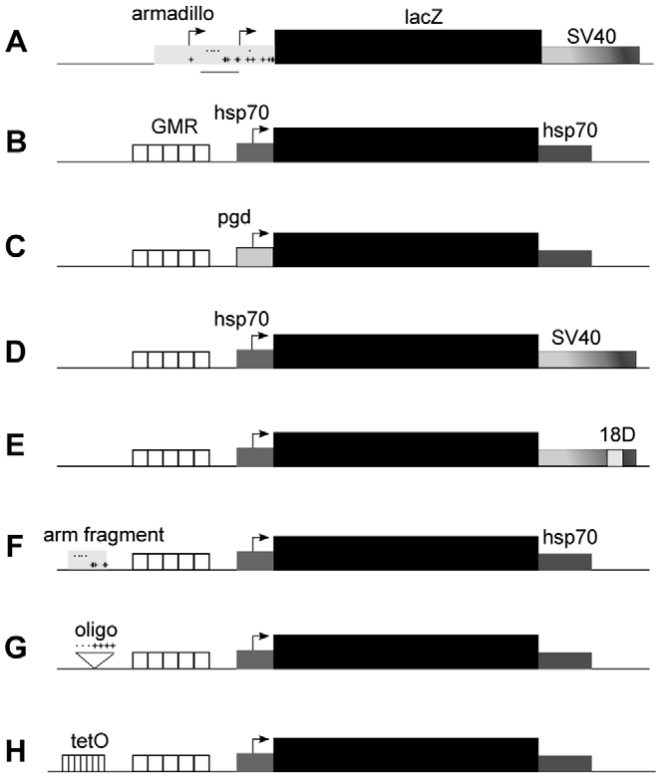
Transgenic *lacZ* constructs used in this study. A–H) The *lacZ* ORF is the solid black box, surrounded by 5′ and 3′ regulatory sequences. Transcription start points represented with bent arrows. Unlabelled elements are identical to the construct immediately above. A, F, G) Sequences identical to, or one mis-match from, the DRE consensus sequence WATCGATW [82], and GAGA (or TCTC), are indicated with points and + symbols, respectively. F) The fragment from the *armadillo* promoter included in arm*-GMR-lacZ is underlined in panel A.

To more quantifiably measure compensation of a GMR-driven transgene, we used the enhancer-promoter to drive the beta-galactosidase gene *lacZ*, and compared beta-galactosidase activity from several related constructs (Figure 2). To measure the degree of up-regulation and compensation supported by GMR-mediated expression, we compared the response of *GMR-lacZ* at X-linked sites to that of *lacZ* driven by the constitutive promoter from the X-linked *armadillo* (*arm*) gene. We have previously shown that X-linked insertions of *arm-lacZ* generally acquire dosage compensation [24], [42]. To remove position effects of integration, we used the phiC31 recombinase system to target transgenic constructs to defined *attP* landing sites [43]. We tested five X-linked landing sites [44] for efficiency of transformation, but could not generate insertions at attP-3B, and found that insertions at attP-3Aa were either male-lethal or ectopically integrated. Of the three remaining sites, attP-2A and attP-6E were within 20 kb of MSL binding sites noted in MSL immuno-precipitates [19], and the peri-centromeric attP-20C was devoid of bound MSL. Flies carrying *GMR-hid* at each of these locations had eyes similar to those with *P*-element insertions of the transgene (Figure 1A, lines C70, C72, C74).

**Figure 3:**
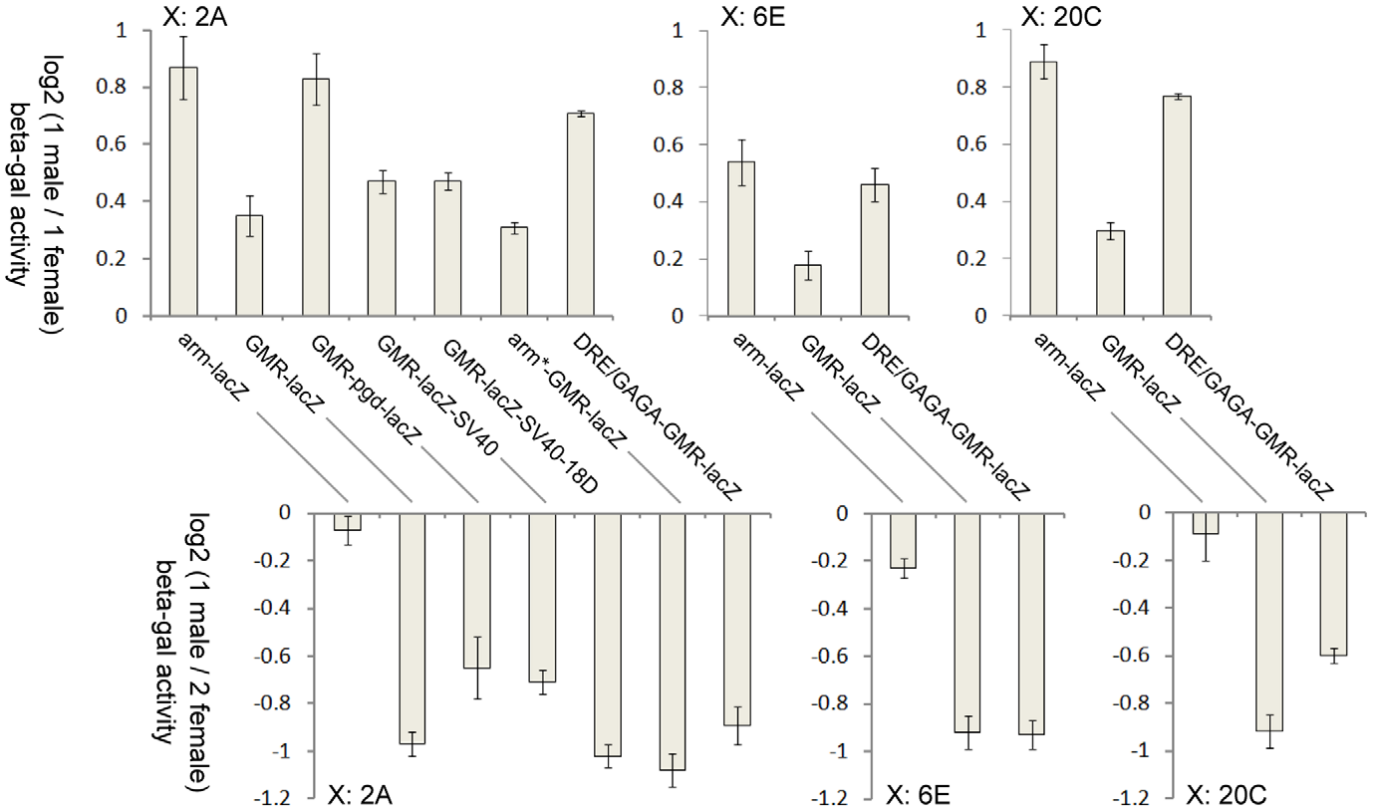
The promoter affected compensation of *lacZ*-mediated beta-galactosidase activity. beta-galactosidase activity was measured in transgenic males and females carrying the indicated *lacZ* constructs at defined attP landing sites (2A, 6E, 20C) on the X chromosome. Top panels: Hemizygous male activity was compared to heterozygous female activity to measure level of male hyperactivation (A 2-fold increase  =  log_2_ of 1). Bottom panels: Hemizygous male activity was compared to homozygous female activity to measure efficiency of dosage compensation (complete compensation  =  log_2_ of 0). Means of triplicates were plotted, with 1 standard error.

We targeted both *arm-lacZ* and *GMR-lacZ* to each of the three X-linked *attP* sites, and measured beta-galactosidase activity (Table 1). We compared activities in (hemizygous) males to those in females heterozygous for the insert, as a measure of male hyper-activity (Figure 3, top panels). Separately, we compared males to homozygous females, to measure dosage compensation (Figure 3, bottom panels). We took the log_2_ of the ratios, to enable a more accurate comparison of ratios above and below zero. A two-fold increase would give a log_2_ of 1.0, whereas equal expression would give 0. As expected, *arm-lacZ* males had nearly twice the activity of single-copy females (top panels), nearly sufficient to compensate for deficiency against two-copy females (bottom panels), although the response at 6E was slightly lower. However, male activity in lines with *GMR-lacZ* was only slightly above single-copy female activity, providing effectively no dosage compensation against two-copy females. The apparent discrepancy between the two ratios (slight hyper-activity but no dosage compensation) is explored below. As the *lacZ* sequences were identical between *arm-lacZ* and *GMR-lacZ*, these results imply that the GMR-*hsp70* regulatory sequences were responsible for poor dosage compensation.

**Table 1:** beta-galactosidase activity of lines carrying *lacZ* constructs at differing *attP* sites.

			Heterozygous (1 dose)				Homozygous (2 doses)			
	attP		Activity^1^		Male/Female		Activity		Male/Female	
Construct	Location		Male	Female	Ratio	log_2_(Ratio)	Male^2^	Female	Ratio	log_2_(Ratio)
arm-lacZ	X	2A	0.35±0.03^3^	0.20±0.03	1.84±0.14	0.87±0.11	0.31±0.02	0.32±0.01	0.95±0.04	−0.07±0.06
		6E^4^	0.50±0.04	0.35±0.02	1.46±0.08	0.54±0.08	0.43±0.02	0.50±0.02	0.85±0.02	−0.23±0.04
		20C^4^	0.47±0.05	0.26±0.03	1.86±0.07	0.89±0.06	0.42±0.01	0.44±0.02	0.95±0.07	−0.09±0.11
GMR-lacZ	3	86F	2.46±0.13	2.05±0.03	1.20±0.06	0.26±0.07	6.28±0.36	4.89±0.05	1.28±0.08	0.36±0.09
	X	2A	2.35±0.07	1.85±0.04	1.28±0.06	0.35±0.07	1.28±0.05	2.52±0.11	0.51±0.02	−0.97±0.05
		6E	1.81±0.11	1.60±0.14	1.13±0.04	0.18±0.05	0.95±0.02	1.79±0.08	0.53±0.01	−0.92±0.04
		20C	2.11±0.02	1.72±0.04	1.23±0.03	0.30±0.03	1.13±0.06	2.14±0.11	0.53±0.03	−0.92±0.07
GMR-pgd-lacZ	X	2A	0.089±0.002	0.050±0.002	1.79±0.11	0.83±0.09	0.130±0.005	0.204±0.011	0.64±0.05	−0.65±0.13
GMR-lacZ-SV40	X	2A	4.07±0.07	2.93±0.07	1.39±0.04	0.47±0.04	5.72±0.28	9.32±0.38	0.61±0.02	−0.71±0.05
GMR-lacZ-SV40-18D	X	2A	5.38±0.12	3.88±0.09	1.39±0.03	0.47±0.03	4.59±0.37	9.31±0.54	0.49±0.02	−1.02±0.05
arm*-GMR-lacZ	X	2A	2.08±0.02	1.68±0.01	1.24±0.01	0.31±0.02	2.02±0.11	4.29±0.23	0.47±0.02	−1.08±0.07
DRE/GAGA-GMR-lacZ	3	86F	0.29±0.01	0.28±0.01	1.05±0.01	0.07±0.02	0.90±0.03	1.00±0.03	0.91±0.05	−0.14±0.08
	X	2A	0.353±0.005	0.215±0.002	1.64±0.01	0.71±0.01	0.293±0.011	0.545±0.031	0.54±0.03	−0.89±0.08
		6E	0.46±0.02	0.33±0.01	1.38±0.05	0.46±0.06	0.73±0.01	1.39±0.05	0.52±0.02	−0.93±0.06
		20C	0.074±0.002	0.043±0.001	1.71±0.01	0.77±0.01	0.090±0.010	0.136±0.016	0.66±0.01	−0.60±0.03

1 OD/min/mg protein.

2 X-linked males hemizygous; only one dose of transgenic cassette.

3 All data are means of triplicates ± standard error.

4 Measured in hemisected adults; all others, adult heads.

Why did *GMR-lacZ* fail to compensate? Perhaps the construct actively blocked the compensatory machinery, although no such elements have yet been reported. Alternatively, the construct may have lacked some feature necessary for transcription to be compensated. We selected the landing site at 2A, which supported strong expression and rates of transformation, and tested the effect of a variety of modifications to *GMR-lacZ* on the male hyper-activity and dosage compensation. Since the MSL complex binds to the body of active X-linked genes with a bias towards the 3′ end, we first considered replacing the 3′ *hsp70* sequences with those from SV40 that were used for *arm-lacZ*. The *GMR-lacZ-SV40* line produced much higher levels of beta-galactosidase than *GMR-lacZ*, possibly because the *lacZ* RNA is less stable with *hsp70* 3′ UTR sequences [45]. There was also a small increase in male hyper-activity and dosage compensation, in comparison to *GMR-lacZ* but the increase was not statistically significant. To enhance binding of the MSL complex to the 3′ end of the transgene, we inserted of an MRE (from 18D11) in the SV40 3′ UTR of *GMR-lacZ-SV40*. However, we found no measureable effect on male hyper-transcription (Table 1).

We next considered that the *GMR-hsp70* enhancer-promoter lacked elements that are present in the *arm* promoter and are required to recruit/respond to the MSL complex. The *arm* promoter fragment contains short runs of GA, similar to the binding sites for the GAGA factor [46] (Figure 2). The GAGA factor has itself been linked to dosage compensation, as it genetically interacts with *msl* mutations to alter male lethality [47]. We also identified a cluster of four DRE-like elements in the *armadillo* promoter. When analysed on a cDNA array, DRE is over-represented in fragments bound by MSL1 immuno-precipitates, compared to those not bound (28 and 16%, respectively), and is slightly enriched on the X chromosome [18], implying a possible role in dosage compensation. The *arm* promoter does not contain an MRE. However, an MRE is found within the gene, but this is downstream of the promoter fragment used in *arm-lacZ* (A. Alekseyenko, personal communication). We isolated a short fragment of the *armadillo* promoter (arm*) spanning the cluster of DRE-like elements and five GAGA-like elements, and tested it upstream of *GMR-lacZ*. The fragment did not include the transcription start site. The activity levels and sex ratio from arm*-GMR-lacZ were similar to those in flies without the *arm* fragment (Table 1). We next tested an oligonucleotide containing four consecutive GAGA sites and three DRE sites, designed to mimic sequences known to bind GAGA factor and DREF in vitro [48], [49]. The activity of *DRE/GAGA-GMR-lacZ* was lower in both sexes than *GMR-lacZ* alone, suggesting that the DRE and/or GAGA elements have inhibited GMR-mediated transcription enhancement. However, the male hyper-activity was significantly higher than for *GMR-lacZ* (P<0.05, T-test).

We expanded on these observations with *DRE/GAGA-GMR-lacZ*. We targeted the construct to the *attP* landing sites and 6E and 20C, and found similar responses of the transgene at all locations. We checked that the observed increase was specific to the X chromosome, by targeting the construct to an autosomal *attP* (at 86F). Indeed, males and females with *DRE/GAGA-GMR-lacZ* at 86F had similar activity levels (Table 1). We crossed the *DRE/GAGA-GMR-lacZ* 2A line to an *hsp83-msl2/Sb* line, and found that female offspring with *hsp83-msl2* had a significantly higher beta-galactosidase activity (P<0.05, T-test) than those without (Table 2); a response to induced MSL complex equal in magnitude to that of *arm-lacZ*. This argues that the male-specific increases observed above were likely due to the MSL complex.

**Table 2:** Effect of *hsp83-msl2* on female beta-galactosidase activity.

	Activity^1^		*Sb^+^/Sb* 2	
Construct	*lacZ/+; hsp83-msl2*	*lacZ/+; Sb*	Ratio	log_2_(Ratio)
arm-lacZ	0.277±0.0213	0.219±0.005	1.27±0.10	0.33±0.11
GMR-lacZ	2.52±0.14	2.69±0.08	0.94±0.04	−0.10±0.07
DRE/GAGA-GMR-lacZ	0.65±0.03	0.51±0.03	1.29±0.01	0.37±0.01

1 OD/min/mg protein.

2 *w;* +*/CyO; P{w^+^ = hsp83-msl2}/ TM6C, cu* ^1^ *Sb* ^1^ males were crossed to females carrying the appropriate *lacZ* cassette at 2A, and Sb and Sb^+^ daughters compared.

3 All data are means of triplicates ± standard error.

Lastly, we considered that the *GMR-hsp70* enhancer/promoter does not respond to the MSL complex because of the *hsp70* minimal promoter. To separate the GMR elements from the *hsp70* sequences, we replaced the *hsp70* TATA with a basal promoter (−42 to +23) from the X-linked dosage compensated *Pgd* gene [50]. The activity from *GMR-pgd-lacZ* was much reduced compared to *GMR-lacZ* in both sexes (Table 1), which was not unexpected given that the *hsp70* promoter has a relatively high basal activity at 25°C [51]. However, male hyper-activity was significantly higher than for *GMR-lacZ* (P<0.05, T-test) and dosage compensation appeared to be partially restored.

### Hyper-activity of *GMR-lacZ* in homozygous females

The two methods for comparing male and female activity of all GMR-based constructs appeared to give slightly different results. Constructs with very low hyper-transcription (e.g. *GMR-lac*Z, arm*-GMR-lacZ) had no dosage compensation, and those with high male hyper-transcription (*GMR-pgd-lacZ*, *DRE/GAGA-GMR-lacZ*) had only modest dosage compensation. Because (hemizygous) males in each measurement were genetically identical, this anomaly can only be explained by differences in female expression. Due to experimental variation, the raw male or female activities cannot be directly compared without first controlling for variation by normalization, as with a male to female ratio. But if we instead calculate the inverse (female to male) ratios, then we have comparable activity estimates of females, normalized to single-copy males (Table 3). As expected, females carrying two copies of *arm-lacZ* had about twice the activity of single-copy females, which is why a male hyper-activity of two-fold (log_2_ of 1.0) is sufficient to dosage compensate. But females with two copies of all GMR-based constructs have more than twice the activity of single-copy females. This explains the discrepancy: a slight male hyper-activity was only sufficient to equal about half the activity of paired females, and full compensation would require male hyper-activity of around 2.5 to 3.0-fold (log_2_ of 1.3–1.6). The significance of this homozygous effect is discussed below.

**Table 3:** Increase in female head beta-galactosidase activity when homozygous for the *lacZ* transgene.

		Female Activity^1^		Homozygous
Construct	Location	One Dose	Two Doses	Increase
arm-lacZ	2A	0.55±0.04	1.05±0.04	1.91
	6E^2^	0.69±0.04	1.17±0.03	1.70
	20C^2^	0.54±0.02	1.07±0.08	1.98
GMR-lacZ	2A	0.79±0.04	1.97±0.06	2.49
	6E	0.88±0.03	1.89±0.05	2.15
	20C	0.81±0.02	1.90±0.09	2.35
GMR-pgd-lacZ	2A	0.54±0.05	1.58±0.15	2.82
GMR-lacZ-SV40	2A	0.72±0.02	1.63±0.06	2.26
GMR-lacZ-SV40-18D	2A	0.72±0.01	2.04±0.08	2.83
arm*-GMR-lacZ	2A	0.81±0.01	2.13±0.11	2.63
DRE/GAGA-GMR-lacZ	2A	0.61±0.01	1.86±0.11	3.05
	6E	0.73±0.03	1.91±0.07	2.62
	20C	0.586±0.005	1.514±0.031	2.56

1 Mean OD/min/mg protein, normalized to single dose male activity, ± standard error, n = 3.

2 Measured in hemisected adults; all others, adult heads.

### Early or constitutive expression of *GMR-lacZ* is insufficient to lead to compensation

It was also possible that *GMR-lacZ* failed to compensate because of problems with expression time or specificity. Perhaps dosage compensation was less efficient in the developing eye tissue. The set of MSL-bound genes in larval salivary glands appears to be a subset of the total MSL-bound genes in early embryos, prompting the suggestion that MSL complex may bind early, and maintain largely stable binding through development [18]. The same conclusion was reached after observing that MSL complex can colocalize with RNA polymerase II on salivary gland chromosomes, but remain bound after polymerase dis-associates [52]. We next tested the relative importance of early and constitutive expression on the late, tissue-specific compensation of *GMR-lacZ*.

We placed the *tetO* tetracycline operator upstream of *GMR-lacZ* to allow additional regulation through the tetracycline system [53]. DNA binding of the tetracycline-controlled transcriptional activator (tTA) is inhibited if tetracycline is added to the culture media (i.e. a tet-OFF system). Early expression of tTA was driven by promoters from the *nullo* and *serendipity alpha* genes [54]. *nullo* and *serendipity alpha* encode proteins that are required for cellularization and are predominately expressed in the early stages of embryogenesis [55]. Expression from each activator (*n1-tTA* and *s1-tTA*, respectively) is sufficient to lead to cell death through expression of a *tetO-hid^ala5^* transgene, but only in the absence of tetracycline [54]. *arm-tTA* [56] was used as a constitutive driver of tTA.

**Figure 4:**
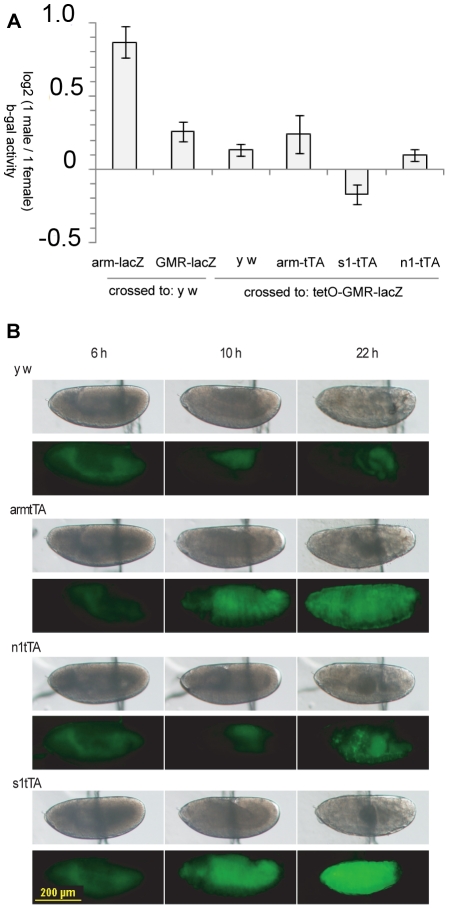
Compensation of *tetO-GMR-lacZ* was unaffected by additional early or constitutive expression. A) Equality of beta-galactosidase activity between the sexes was measured in adult heads of flies carrying one copy of *tetO-GMR-lacZ* (at the 2A attP site), and one copy of the indicated tetracycline driver. *y w* flies have no tTA driver. The responses of *arm-lacZ* and *GMR-lacZ* at 2A are provided for comparison. Flies were raised in the absence of tetracycline to promote transcription activation by tTA. Means of triplicates were plotted, with 1 standard error. B) Activity of the tTA drivers was detected as yellow fluorescence (through a green filter set) in embryos carrying one copy of tetO-YFP, and one copy of the indicated drivers, raised without tetracycline. White light (top of each pair), and fluorescent images of the embryos at different time points after egg laying were recorded. Dorsal up, anterior left. The solid line bisecting each embryo is the edge of tape used to mount the embryos.

We crossed flies carrying *tetO-GMR-lacZ* to lines with *arm-tTA*, *n1-tTA*, or *s1-tTA* activators, and raised the offspring in the absence of tetracycline. We measured the male hyper-activity of beta-galactosidase in adult heads (Table 4), and compared the responses to those of *arm-lacZ* and *GMR-lacZ* (Figure 4A). Males and (single-copy) females of all crosses had very similar activities. The response to additional constitutive expression provided by *arm-tTA* was evident in the very high activities in offspring of this cross. Further, the increase in *lacZ* expression was dependent on tTA as there was no response when flies were raised with tetracycline (mean male activity of 2.15±0.06, female of 1.64±0.07 OD/min/mg). A separate cross to *y w* flies (parental strain), which lack tTA, showed that the tetO sequences alone had little effect on activity from *GMR-lacZ* (Table 4). To confirm that the tTA drivers were active in the early embryo, we crossed the tTA driver lines to a *tetO-YFP* responder line [57] and examined the offspring for yellow fluorescence (Figure 4B). We found that all tTA drivers were effective at inducing YFP expression well above background levels.

**Table 4:** Effect of tTA drivers on beta-galactosidase activity from *tetO-GMR-lacZ.*

Cross		Activity^1^		Male/Female	
*lacZ*	x Driver	Male	Female	Ratio	log_2_(Ratio)
*tetO-GMR-lacZ*	x (*y w*)	1.53±0.05^2^	1.40±0.08	1.10±0.03	0.13±0.04
	x s1-tTA	1.70±0.04	1.91±0.06	0.89±0.04	−0.17±0.07
	x n1-tTA	1.74±0.03	1.62±0.03	1.07±0.03	0.10±0.04
	x arm-tTA	8.70±0.53	7.35±0.20	1.19±0.10	0.24±0.13

1 OD/min/mg protein.

2 All data are means of triplicates ± standard error.

**Figure 5:**
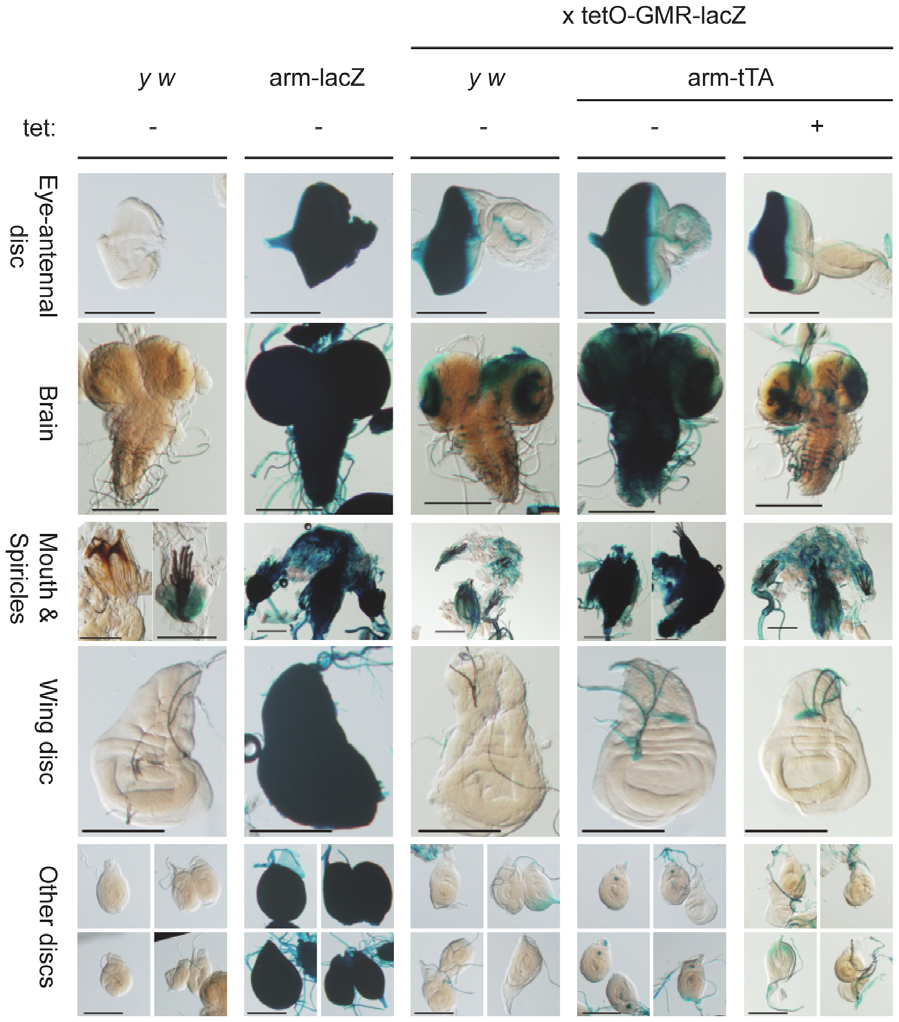
*tetO-GMR-lacZ* responded to tTA in brain and other tissues but not imaginal discs. Third instar larvae of the indicated strains, raised with (+) or without (−) tetracycline (tet) in the diet, were dissected and stained with X-gal for *lacZ* expression. Additional induction of *tetO-GMR-lacZ* occurs with *arm-tTA* in the absence of tetracycline. *y w* and *arm-lacZ* provided as negative and positive controls for *lacZ* expression, respectively. All staining and photographic conditions were equal across all sets. Bar  = 0.2 mm.

To confirm that regulation via the tetracycline system was capable of shifting the expression pattern of *tetO-GMR-lacZ*, we crossed the reporter line to *arm-tTA* flies and observed the altered expression pattern in climbing third instar larvae (Figure 5). The GMR enhancer/promoter is active in all cells in, and posterior to, the morphogenetic furrow in the developing eye-antennal disc in third instar larvae [6]. In a control cross to *y w* flies, beta-galactosidase staining could be detected in the developing eye, posterior to the morphogenetic furrow, and to a limited extent in the brain, consistent with Glass expression [30]. When crossed to *arm-tTA*, beta-galactosidase could also be detected in the brain and most other larval tissues (Figure 5). This response was specific to *arm-tTA* as it was inhibited with the addition of tetracycline to the culture medium. Given that the *tetO-GMR-lacZ* reporter could respond to additional regulation through the tetracycline system, and that the activators were all active in the early embryo, we concluded that the above test of effect on dosage compensation was valid. Therefore, additional early or constitutive expression of *GMR-lacZ* was insufficient to affect dosage compensation. The time and specificity of expression may have been relatively less important than the nature of the promoter/enhancer.

However, the staining for beta-galactosidase (Figure 5) revealed a surprising observation. The pattern of staining seen with *arm-tTA* activation of *tetO-GMR-lacZ* did not equal the pattern from the *arm-lacZ* positive control line. The *tetO-GMR-lacZ* reporter was widely expressed with the notable exception of the developing imaginal discs, beyond the Glass-expressing areas of the eye-antennal disc. As the fragment of *armadillo* was the same in *arm-tTA* and *arm-lacZ* [56], it appears that *tetO-GMR-lacZ* was being repressed in these tissues. These results suggest that the 29 bp GMR enhancer contains the binding site for a repressor that is being expressed in imaginal cells.

## Discussion

Our attempt to screen for modifiers of dosage compensation was thwarted by the failure of *GMR-hid* to be well compensated. The transgene did not acquire compensation in an X chromosome environment, nor when coupled to strong MSL attractors. Quantitative measurements with the *lacZ* gene confirmed that the *GMR-hsp70* enhancer-promoter was at fault. We believe that this is not due to lack of local MSL complex, but rather an inability of the complex to regulate GMR-mediated transcription.

This was most evident in the phenotype of the *GMR-hid* lines with the 9-copy multimer of the *roX1* DHS. In most of these lines, females had a lighter eye color than males, and there was obvious variegation in pigmentation patterns in several lines, suggesting repression of the mini-*white* marker gene. It had been previously shown that recruitment of the MSL complex to a *roX1* transgene led to relief of repression of mini-*white* [41]. Similarly, MSL complex recruited to the *roX1 DHS* in the *(roX1 DHS)9-GMR-hid* lines may have been sufficient to relieve repression on mini-*white*. However, there was little difference in eye size, suggesting that *GMR-hid* was not responding to the bound MSL complex. Lack of response to MSL complex is also consistent with our observations that strong MSL binding sites had no effect on *GMR-lacZ* beta-galactosidase activity. While formally possible that local MSL complex was limiting at each site, the large number of lines screened makes this unlikely. Certainly local MSL complex at the 2A, 6E, and 20C *attP* sites was sufficient to up-regulate *arm-lacZ*, yet had no effect on *GMR-lacZ*. The most likely explanation is that expression from the *GMR-hsp70* enhancer-promoter was not responsive to MSL complex.

Our observation that the promoter can affect dosage compensation was somewhat surprising. The MSL complex binds the male X chromosome most strongly at 150 to 300 mostly non-coding GA-rich MRE sequences [38], [39] but is also highly enriched across most active X-linked genes, with a bias towards the 3′ end [17], [18], [19]. It has been proposed that the complex initially binds to the MRE sites and then spreads to active genes [38]. In support of this model, the MSL complex is attracted to ectopically-transcribed portions of the X chromosome, created when random insertions of a *UAS-hsp70 TATA* transgenic promoter are induced with GAL4 [58]. Further, the complex is recruited to transgenic autosomal insertions of the X-linked *mof* gene, but only when *mof* is transcribed [59]. As recruitment still occurs when *mof* is transcribed from a *tubulin* promoter, GAL4 UAS sequences, or even in an antisense direction, Kind and Akhtar conclude that “the type of promoter and direction of transcription are not [important]”. It appears that some feature of transcription itself, or an active chromatin state, may be responsible for recruitment of MSL complex to the many genes on the X chromosome. This may involve the chromo domain of MSL3, which recognizes histone H4 monomethylated at lysine 20 [60], [61], a marker for active genes in human cells [62]. Accordingly, autosomal genes transposed to the X chromosome acquire MSL binding, H4K16 acetylation, and dosage compensation, but only when the transposed genes are transcribed [63]. However, transcription was disabled by deleting promoter regions. Our observation that *GMR-lacZ* failed to acquire compensation, at the same X-linked sites where *arm-lacZ* did, argues that transcription alone is not sufficient for a gene to be compensated, and that the importance of a gene's promoter in dosage compensation cannot be ignored. Indeed, studies of the X-linked *white* gene concluded that the gene contains multiple elements that influence dosage compensation, some near the promoter and some in the coding region [64], [65]. Further, it has been suggested that DNA supercoiling factor bound at promoter regions may facilitate loading of the MSL complex onto active genes [33]. DNA supercoiling factor contributes to *Drosophila* dosage compensation [10].

Our efforts to determine why genes driven by the GMR enhancer-promoter do not respond to the MSL complex were complicated by the surprising observation that activity in homozygous females was more than twice that of hemizygous females. We believe this may be because of a complex interplay between the Glass transcription factor and an unknown repressor. As a consequence of the hyperactivity in homozygous females, the comparison of the activity of one dose males with one dose females provides a more accurate view of male hyperactivation. Of the various gene constructs evaluated, two showed a significant increase in activity in males. In one construct, the *hsp70* basal promoter was replaced with the weaker basal promoter from the X-linked dosage compensated *Pgd* gene. This suggests that the *Pgd* basal promoter contains elements that are important for recruitment of the MSL complex to the reporter gene and that the *hsp70* basal promoter lacks these elements. The *white* gene promoter was concluded to be important for dosage compensation because autosomal *white* transgenes driven by the full heat-inducible *hsp70* promoter were expressed equally in males and females [64]. Autosomal *white* transgenes are typically expressed at higher levels in males than females [66]. An alternative interpretation of these results is that the *hsp70-white* transgene is not compensated because the *hsp70* promoter does not respond to the MSL complex. The *Pgd* promoter did not contain any obvious binding sites for known transcription factors. Widely expressed genes such as *Pgd* and *arm* generally contain similar levels of RNA polymerase II at 5′ and 3′ regions of the transcription units [67]. It has been suggested that RNA polymerase II stalling at 5′ ends depends upon core promoter elements that are absent in constitutively expressed genes [67]. Thus it may be of interest to determine if the MSL complex can only regulate those X-linked genes that do not have stalled promoters. The other construct that showed significant increased activity in males contained binding sites for the GAGA and DREF DNA binding proteins upstream of the GMR-*hsp70* enhancer-promoter. The DREF and/or GAGA factors could create a local chromatin environment that facilitates recruitment of the MSL complex or could interact directly with the complex. It may now be beneficial to separate the effects of DRE and GAGA elements, and determine such characteristics as motif strength, placement, and repeat spacing. Such studies may explain why addition of a fragment from the *arm* promoter that contained predicted GAGA and DREF sites, did not lead to a significant increase in *GMR-lacZ* expression in males. We found no support for the notion that late or tissue-specific expression was a factor in poor compensation, although it is possible that these factors affect compensation of other genes. Thus we conclude that genes driven by the GMR-*hsp70* enhancer-promoter do not respond to the MSL complex because of the nature of the *hsp70* basal promoter and because it lacks elements such as GAGA and DREF binding sites.

A curious possibility is that any one of the promoter modifications could have caused or increased repression of the transgene, perhaps via the putative repressor detected in larval staining for beta-galactosidase. This may be reflected in the generally lower beta-galactosidase activity in lines with *pgd-GMR-lacZ* and *DRE/GAGA-GMR-lacZ* transgenes. Knowing that MSL complex can act to relieve repression in males, such changes would also register in our tests as an apparent increase in male hyper-activity. However, we cannot distinguish between general repression with male-specific relief, and more simple male enhancement, as our assays were designed to measure the final result of dosage compensation.

Can our results shed any insight into why other X-linked genes are not dosage compensated? The non-compensated X-linked *Lsp1alpha* gene has been particularly well studied [42], [68], [69]. *Lsp1alpha* is not enriched for H4K16ac in the tissue in which it is active [42]. Similarly, the *runt* gene, which is dosage compensated by an MSL-independent mechanism [70], is not enriched for H4K16ac in Drosophila embryos [16]. Thus it appears that these genes are not hypertranscribed by the MSL complex due to a failure to recruit the complex rather than because their promoters lack certain elements. Indeed, X-linked *lacZ* transgenes driven by the *Pgd* promoter are enriched for H4K16ac [42]. Whether or not this is the general explanation for why some X-linked genes are not compensated would require a more global approach to compile a list of non-compensated genes. To our knowledge no such list exists, however it may be possible to do this using the extensive existing data sets of male vs female expression, MSL2 RNAi knockdown in cell lines and MSL complex binding and H4K16ac enrichment profiles [13], [38], [71], [72]. However, in general almost all active genes on the male X chromosome are enriched for H4K16ac, which has a broader distribution profile than the MSL complex [13]. Thus since there are very few active X-linked genes that are devoid of H4K16ac, either there are very few non-compensated genes or other factors such as the nature of the gene promoter influence male hypertranscription.

Beyond the promoter, our experiments also revealed that another factor can affect the degree of compensation. Two copies of all GMR cassettes had more than twice the beta-galactosidase activity of one copy, instead increasing three- to four-fold. As we only measured this effect in females with X-chromosomal insertions, it would be interesting to extend the experiments to autosomes and males. The non-linear effect did not occur with *arm-lacZ*, and is not a property of the beta-galactosidase assay, which is linear across a 100-fold range of protein extract concentration [51]. As a similar effect occurred in all cassettes with GMR, regardless of the surrounding sequences, the effect appears to be due to paired GMR enhancer elements. Homologous chromosomes in *Diptera* pair through all mitotic stages, not just during meiosis [73]. Paired homologues can share transcription factors or chromatin regulators, which can affect transcription in a variety of ways known as ‘transvection’ [74]. The pairing effect of GMR may similarly reflect synergy in the binding or activity of positive factors such as Glass. Conversely, negative regulators such as the putative repressor could have less effect on the paired GMR elements than would be expected for two un-paired elements. We attempted to determine if the non-linear response was pairing-dependent by measuring two copies of *GMR-lacZ* cassettes at different locations, but found that the differing activity levels at each location precluded conclusions based on mixed genotypes. Whatever the cause, the non-linear effect has consequences for dosage compensation of *GMR-lacZ*, as male expression needs to be more than doubled to equal the paired female levels.

More generally, a description of X chromosome dosage compensation as a transcriptional doubling by the MSL complex may be too simple. When quantified by microarray, X chromosomal transcripts reduced a variable amount after knock-down of *msl-2*, with median decreases of about 1.3 to 1.7-fold [72], [75]. Recent estimates based on high-throughput sequencing of RNAs in S2 cells put the effect of MSL-mediated up-regulation at about 1.4-fold [71]. Zhang et al. believe that the remaining up-regulation required to equalize gene dose is likely provided by a separate mechanism that also compensates for aneuploid regions genome-wide. Gene expression in aneuploid regions of S2 cells, or in chromosome aberrations, can be “buffered” to varying extents, with levels closer to wild-type than would be expected from gene copy number [71], [76], [77]. If *Drosophila* males have indeed evolved to co-opt gene buffering as a part solution to X chromosomal haploidy, then dosage compensation should be viewed as a combinatorial process. At the level of an individual X-linked gene, male expression may be affected by any or all of several mechanisms, including aneuploidy-type buffering, dose-dependent regulatory feedback, and MSL-mediated up-regulation. Given that the paired female X-linked genes might also be affected by transvection, dosage compensation could be seen as merely the result of a combination of mechanisms. Care must be taken in the design of experiments to ensure the correct biological process is measured.

Finally, it should be noted that the tissue-specificity of the *GMR* enhancer appears to reflect more than simple activation by the Glass transcription factor. We observed a general lack of GMR-lacZ expression in larval imaginal tissues beyond those that express Glass. This repression pattern is similar to that observed for another Glass reporter, “38-1”, a pentamer of a larger (38 bp) fragment from the *Rh1* proximal enhancer spanning the Glass binding site [30]. 38-1 cannot respond to *glass* overexpression (from a *hsp70-glass* transgene) in imaginal tissue with the exception of a small number of cells in the leg disc. In contrast, in response to *glass* overexpression, 38-1 directs *lacZ* expression in many cells in the brain and central nerve cord. A shorter 29 bp enhancer (GMR) responded more strongly to *glass* overexpression, directing *lacZ* expression in many cells in the brain and in all cells of the eye/antennal and leg discs. It was concluded that shortening the enhancer to 29 bp had deleted the binding site for a putative repressor that inhibits Glass activity in non-photoreceptor cells [30]. DNA sequencing of *GMR-lacZ* used in this study confirmed that we were using the pentamer of the 29 bp element. One explanation for our results is that the repressor binding site was not completely removed when shortening the fragment from 38 to 29 bp, merely weakened to allow dominance of high levels of Glass over the repressor. Alternatively, the 38 bp enhancer contains binding sites for two repressors, one of which is retained in the 29 bp GMR enhancer. Additional studies would be required to distinguish between these two possibilities. In either case, it appears that the tissue-specific expression directed by the 29 bp GMR enhancer is due to combined action of the Glass activator and an unknown repressor.

## Materials and Methods

### Recombinant DNA

Standard molecular cloning techniques [78] were used, with clones confirmed by restriction analysis, PCR, or DNA sequencing.

#### *hid* gene constructs

An EcoRI to EcoRI fragment of pBS SK *hid* cDNA [7], encoding *hid* cDNA, was inserted into the EcoRI site of pGMR-1 [4] to create pGMRhid, which contains the *GMR-hid* cassette used for most *GMR-hid* lines. A XhoI to NotI fragment of pCaSpeR3 *roX1* DHS short or pCaSpeR3 *roX1* DHS short multimer [26], containing the *roX1* DHS or its multimer, was inserted into the XhoI site of pGMR-1, with the aid of oligonucleotides (5′-TCGACGTTTAAACGGTTGGCC-3′ and 5′-AATTGGCCAACCGTTTAAACG-3′) annealed to link EcoRI and XhoI sites, to create pCL02 or pCL03, respectively. A EcoRI to EcoRI fragment of pBS SK *hid* cDNA containing *hid* was inserted into the EcoRI site of pCL02 or pCL03, to create pRGH and pR9GH, which contained the *roX1 DHS-GMR-hid* or *(roX1 DHS)9-GMR-hid* cassettes, respectively. pCaSpeR3 18D10-L monomer or pCaSpeR3 18D10-L4mer [40] was digested with NotI, treated with Klenow fragment, then digested with BamHI, and the fragment encoding the 18D site, or its multimer, was inserted into the BglII and StuI sites of pGMRhid to created p18DGH, and p18D4GH, which contain the *GMR-hid-18D* and *GMR-hid-(18D)4* cassettes, respectively. An EcoRI to EcoRI fragment of pTAattB [43], encoding *attB*, was treated with Klenow fragment and inserted into the EcoRV and SmaI sites of pBluescript II KS+ (Invitrogen) to create pBSattB. pBSattB was partially digested with XhoI and re-ligated to remove the XhoI site, then a HindIII to HindIII fragment of pGMR-1, encoding mini-*white* and GMR, was inserted into the HindIII site to create pBSw+GMRattB. An EcoRI to EcoRI fragment of pBS SK hid cDNA, encoding *hid* cDNA, was inserted into the EcoRI site of pBSw+GMRattB to create pGHattB, which contains the *GMR-hid* cassette used for phiC31-mediated transformations (lines C70, C72, C74).

#### *lacZ* constructs

An EcoRI to EcoRI fragment of pTAattB, encoding *attB*, was inserted into the EcoRI site of pCaSpeR-arm-betagal [79], to create pALattB, which contains the *arm-lacZ* cassette. pCaSpeR-arm-betagal was digested with XbaI, treated with Klenow fragment, and digested with SmaI, then the fragment encoding *lacZ* was inserted into pBC KS + (Invitrogen) to create pBClacZ. A BamHI to EcoRI fragment of pBClacZ, encoding *lacZ*, was inserted into the BglII and StuI sites of pBSw+GMRattB to create pGLattB, which contains the *GMR-lacZ* cassette. To make pGMR-pgd-lacZ, pGLattB was digested with XbaI (partial) and EcoRI and the excised *hsp70* minimal promoter was replaced with a 70 bp linker that contained the *Pgd* minimal promoter. The 70 bp linker was made by annealing the oligonucleotides 5′-CTAGAACAGTGCCATATATAGATTGTAACATTAGGAGCTCAAATCATTGTTGGAACACAAACCACAAAG-3′ and 5′-AATTCTTTGTGGTTTGTGTTCCAACAATGATTTGAGCTCCTAATGTTACAATCTATATATGGCACTGTT-3′. pGL-H was made by digestion of pGLattB with HindIII (partial) and re-ligation to remove the site in the pUC backbone. A SmaI to HindIII fragment of pCaSpeR-arm-betagal, encoding *lacZ* and SV40 3′ UTR, was inserted into the EcoRI (filled) and HindIII sites of pGL-H to create pGLSV40attB, which contains the *GMR-lacZ-SV40* cassette. A SmaI to HindIII fragment of pBS2N17merHF12-1x12 [80] encoding *lacZ*, SV40 3′ UTR and the 18D site, was inserted in the (blunted) EcoRI and (remaining) HindIII sites of pGL-H to create pGLSV4018DattB, which contains the *GMR-lacZ-SV40-18D* cassette. A XhoI to XhoI fragment of a PCR amplicon (primers 5′-CCGGAATTCTCGAGTGGAATGTAAACAATGCCACAGAC-3′ and 5′-CCGGAATTCTCGAGTAAACGGAACAGAATCACAGATGC-3′) of the *armadillo* promoter, from pCaSpeR-arm-betagal was inserted in the XhoI site of pGLattB to create pCL12, which contains the arm*-GMR-lacZ cassette. Four oligonucleotides (5′-AATTGCTCGAGCTAGCTATCGATAGATTCCCTGCTATCGATAGATTCCCTGCTATCGA-3′, 5′-TAGATTCGCTAGCAGATCTCTCTCGTTCATTGAGAGAGCAAAGGCCTCTCTCGTTCATTGAGAGAGATCTCGAG-3′, 5′-AATTCTCGAGATCTCTCTCAATGAACGAGAGAGGCCTTTGCTCTCTCAATGAACGAGA-3′, and 5′-GAGATCTGCTAGCGAATCTATCGATAGCAGGGAATCTATCGATAGCAGGGAAATCTATCGATAGCTAGCTCGAGC-3′) were annealed to create 3 DRE and 4 GAGA sites, digested with XhoI, and inserted into the XhoI site of pGLattB to create pCL13, which contains the *DRE/GAGA-GMR-lacZ* cassette. A XhoI-SacI fragment of pW.T.P-2 [53], encoding *tetO*, was inserted with the aid of oligonucleotides (5′-CTTTAAACGGTTGGCG-3′ and 5′-TCGACGCCAACCGTTTAAAGAGCT-3′), annealed to link SacI to XhoI, in the XhoI site of pGLattB to create pCL21, which contains the *tetO-GMR-lacZ* cassette. The linkers are 3′ of the *tetO*.

### *Drosophila* genetics, transformation, image capture and beta-galactosidase assays

To observe *GMR-hid* eye size with *msl* deficiencies, *msl2 msl1 mle/CyO* males were mated to virgin *GMR-hid* C60 females, and Cy and Cy^+^ offspring compared. To observe *GMR-hid* eye size with constitutive expression of *msl2*, virgin *GMR-hid* C60 females were separately crossed to *w; msl1* ^L60^ */CyO; P{*w^+^  =  *hsp83-msl2}* males [36] and *y^1^ w^1^; msl1^L60^/CyO, pr cn^2^ y* ^+^ males. Curly offspring of each cross were compared. To measure *GMR-lacZ* activity with constitutive expression of *msl2*, *w; msl1* ^L60^ */CyO; P{w^+^ = hsp83-msl2}* males were crossed to virgin *w* ^1118^ *; P{w* ^+mC^ *=  hs.hid}3, Dr* ^1^ */TM6C, cu* ^1^ *Sb* ^1^ females, then the resulting curly-winged, stubble-bristled males were mated to virgin females carrying the appropriate *lacZ* cassette at 2A, and Sb and Sb^+^ daughters compared.

Right-hand-side adult eyes that best represented the modal average eye size of each line were illuminated with reflected halogen light. Images of the lines carrying *(roX1 DHS)9-GMR-hid* were captured with a Magnafire camera and software (Optronics), and the RGB colour balance manually altered to best match other images. All other images were captured with an Olympus DP-70 camera, with the provided software or with Soft Imaging System analySIS FiVE 5.0, and balanced RGB on white card to control for exact lighting conditions. The exposure time was automatically determined by the software.

*P*-element mediated transformation of *Drosophila* was performed as previously described [24]. phiC31 recombinase mediated transformation was performed using strains that contained both an *att*P landing site and expressed recombinase in the germ-line [44]. Transgenic flies were back-crossed to the *y w* line, established as homozygous or balanced lines, and confirmed by PCR both within the insert and across the insert/landing site boundary.

Beta-galactosidase assays of hemisected adults or heads were conducted based on the procedure of Simon and Lis [51]. If necessary, tetracycline was added at 10 ug/mL to the media, and flies raised in the dark. New emerged flies were collected and then aged 3 to 5 days at before the assay. For each replicate, 9 females or 12 males were hemisected to remove wings, legs, and abdomens, or 15 female and 20 male heads used. The mean activities of males, females, or ratios between the sexes, are displayed with standard errors (one standard deviation / square root of sample size (3)), with a log_2_ transformation then often applied to allow better comparisons of different ratios. Two sample, two-tailed Student's *t*-tests were conducted in the R software environment, using (the default) Satterthwaite's approximation. Tissue dissected from climbing third instar larvae was stained with X-gal essentially as described by Glaser et al. [81]. The stained tissue was mounted in 90% glycerol, and photographed under transmitted white light at 1/90 second exposure, ISO 200, with an Olympus DP-70 camera.

## Supporting Information

Figure S1 ***GMR-hid*** **did not respond to the MSL complex.** Eyes of flies that carried a single copy of the *GMR-hid* transgene (line C60), and were either wild type for the *msl* genes, heterozygous for *msl1*, *msl2* and *mle* or constitutively expressed *msl2* (*hsp83-msl2*). If *GMR-hid* on the X chromosome responded to levels of the MSL complex then the eye size in *msl1 msl2 mle* heterozygous males should have been larger than control. Similarly, eyes of females that expressed *msl2* should have been smaller than control.
(TIF)
