# Supplementary figures and images for: Abnormal Dosage Compensation of Reporter Genes Driven by the Drosophila Glass Multiple Reporter (GMR) Enhancer-Promoter
Source: PLoS One. 2011 May 31;6(5):e20455. doi: 10.1371/journal.pone.0020455 (PMC3105068; doi:10.1371/journal.pone.0020455)

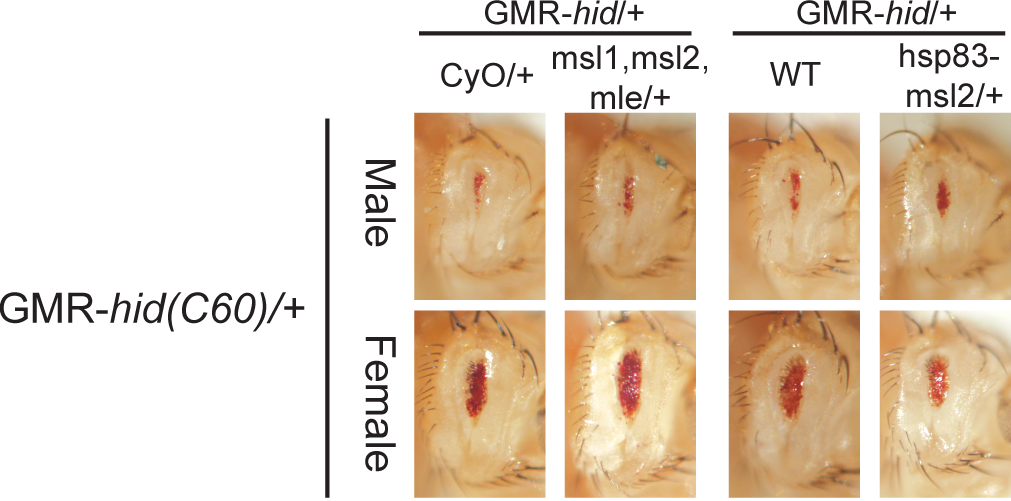

Supplement: Figure S1 — GMR-hid did not respond to the MSL complex. Eyes of flies that carried a single copy of the GMR-hid transgene (line C60), and were either wild type for the msl genes, heterozygous for msl1, msl2 and mle or constitutively expressed msl2 (hsp83-msl2). If GMR-hid on the X chromosome responded to levels of the MSL complex then the eye size in msl1 msl2 mle heterozygous males should have been larger than control. Similarly, eyes of females that expressed msl2 should have been smaller than control. (TIF) [file pone.0020455.s001.tif]
